# Influence of Graft Ureter Length, a Donor-Related Factor, on Urinary Tract Infections After Living-Donor Kidney Transplantation: A Single-Center Analysis of 211 Cases

**DOI:** 10.3389/ti.2022.10754

**Published:** 2022-11-02

**Authors:** Shoma Koga, Shigeyoshi Yamanaga, Yuji Hidaka, Kosuke Tanaka, Akari Kaba, Mariko Toyoda, Shintaro Ochiai, Yuichi Takano, Yasuhiro Yamamoto, Akito Inadome, Hiroshi Yokomizo

**Affiliations:** ^1^ Department of Surgery, Japanese Red Cross Kumamoto Hospital, Kumamoto, Japan; ^2^ Department of Surgery, Graduate School of Medicine, Kyoto University, Kyoto, Japan; ^3^ Department of Nephrology, Japanese Red Cross Kumamoto Hospital, Kumamoto, Japan; ^4^ Department of Urology, Japanese Red Cross Kumamoto Hospital, Kumamoto, Japan

**Keywords:** kidney transplantation, donor-related factors, ureter length, urinary tract infection, ureter diameter

## Abstract

Urinary tract infection (UTI) occurs in 25% of recipients of living-donor kidney transplantation (LDKT). Female sex, age, and anatomical abnormalities have been reported as recipient-related risk factors for UTI after LDKT; few studies have reported donor-related factors. We retrospectively examined UTI occurrence within 5 years of transplantation in recipients (*n* = 211) who underwent LDKT at our hospital between April 2011 and April 2021. All nephrectomies were performed using a retroperitoneal pure laparoscopic approach. The ureter was dissected at the lower level of the common iliac artery and trimmed to the shortest length, enough to reach the bladder using extra vesicular ureterocystoneostomy with a 3 cm submucosal tunnel. Twenty-nine recipients (13.7%) developed UTI within 5 years, and the median time to onset was 40.0 days. After adjusting for the well-known factors, including recipient sex, graft ureter length was an independent factor for UTI occurrence (HR 1.25, 95% CI 1.02∼1.53, *p* = 0.028) in the multivariate Cox regression analysis. The long ureter is usually trimmed, and the widest part is used for anastomosis, which may increase the possibility of reflux from the bladder to the ureter in the standard technique. The ureter length may be associated with the incidence of UTI after LDKT.

## Graphical Abstract

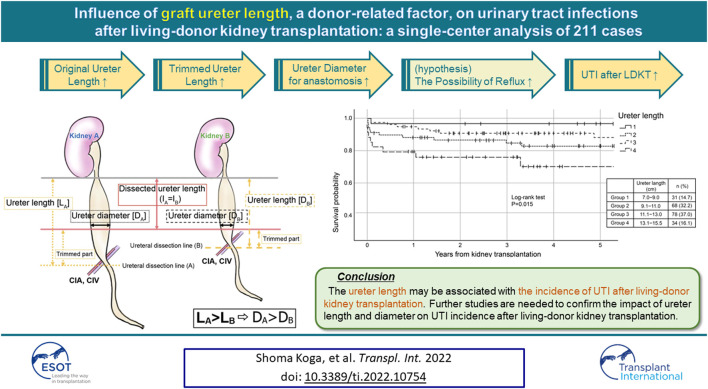

## Introduction

Urinary tract infection (UTI) is one of the most common infections after kidney transplantation. UTI has been reported to occur in 25% of recipients within the first year after living-donor kidney transplantation (1–3). It is associated with increased risks of acute rejection, allograft dysfunction, graft loss, increased duration of hospitalization, and mortality (1, 3–6). Furthermore, recurrent UTI, which occurs in 7% of the patients after kidney transplantation, is one of the leading causes of allograft loss and death (7). Therefore, prediction, early detection, and prevention of UTI is essential.

Recipient-related factors, such as older age, female sex, recurrent UTI before kidney transplantation, number of days with indwelling urinary catheter, congenital urinary tract malformations, vesicoureteral reflux (VUR), history of UTI 1 month before kidney transplantation, and autosomal dominant polycystic kidney disease (ADPKD), are known to increase the incidence of UTI (8, 9). Although there have been many studies regarding the influence of recipient-related risk factors on UTI after kidney transplantation, only a few studies have reported donor-related factors other than deceased donor kidneys (3, 10, 11).

We hypothesized that donor-related factors could also affect the incidence of UTI. We retrospectively examined the association between the incidence of UTI after living-donor kidney transplantation and donor-related factors.

## Materials and Methods

We performed a retrospective analysis of the factors related to UTI occurrence within 5 years of living-donor kidney transplantation. Consecutive 211 recipients who underwent living-donor kidney transplantation at our hospital from April 2011 to April 2021 were included.

A list of recipient and donor characteristics was made according to the previous reports (3, 8–11), and the corresponding information was collected from the electronic medical records. The following recipient characteristics were collected: age, sex, body weight (BW, kg), body mass index (BMI, kg/m^2^), body surface area (BSA, m^2^), presence of diabetes, history of dialysis and duration of dialysis (months), pre-transplant bladder volume (ml). The following donor characteristics were collected: age, sex, weight (kg), BMI (kg/m^2^), BSA (m^2^), graft weight (g), graft volume (cm^3^), graft major axis (mm), graft density (g/cm^3^), graft ureter length (cm), side of the graft (left or right). The ureter length was defined as the length from the lower pole of the kidney to the stump of the ureter. Graft volume and graft density were calculated as follows: graft volume (cm^3^) = Long diameter (mm) x short diameter (mm) x thickness (mm) x 4/3 x π x 1000; graft density (g/cm^3^) = graft weight (g)/graft volume (cm^3^). The eGFR slope (ΔeGFR/year) was calculated as follows: eGFR slope = (the latest eGFR—eGFR at 1-year post-transplant)/(post-operative years of the latest eGFR—1).

### Definition of UTI

We included all recipients with symptomatic uncomplicated and complicated UTI for the analysis. Urinalysis and urine cultures were performed if the recipient had a fever or complained of urinary symptoms.

The definitions of UTI related terms are as follows. Asymptomatic bacteriuria: positive urine culture (identified as >10^5^ colony-forming unit [CFU]) without any symptoms. Uncomplicated UTI (simple cystitis): positive urine culture (identified as >10^5^ CFU) with local urinary symptoms such as dysuria, frequency, and urgency without systemic symptoms such as fever and abdominal pain. Complicated UTI: positive urine culture (identified as >10^5^ CFU) with the systemic manifestation of fever, graft pain, chills, malaise caused by the same bacteria in urine, or biopsy with findings consistent with pyelonephritis(12).

### Immunosuppression Protocols

All patients were administered methylprednisolone (500 mg/body) immediately before graft reperfusion, and basiliximab (20 mg/body) on days 0 and 4. The standard protocol consisted of administration of tacrolimus (TAC), mycophenolate mofetil (MMF), and methylprednisolone. The dosages of TAC and MMF were adjusted to achieve optimal trough levels and area under the curve (AUC) of 0–4 levels as previously reported (13). MMF was started at a dose of 2,500 mg/day when TAC was used and 3,000 mg/day when cyclosporine (CSA) was used from day 1 to day 14; thereafter, MMF was administered at doses of 2,000 mg/day and 1,500 mg/day when TAC and CSA were used, respectively. Methylprednisolone doses were reduced gradually from 60 mg/day on day 0–10 mg/day on day 19 and maintained at 5 mg/day from 6 months after transplantation. Desensitization therapy consisted of rituximab (100∼200 mg/body) twice on day 1 and day 14 or once on day 1, double filtration plasmapheresis four times before kidney transplantation, and MMF (1,000 mg/day) with prednisolone (10 mg/day) from day 14. The intensity of desensitization therapy was determined by the risk-stratified method but modified according to the patient background.

### Operative Methods and Post-operative Managements

All nephrectomies were performed using a retroperitoneal pure laparoscopic approach. The surrounding tissue of the ureter was carefully preserved and dissected at the lower level of the common iliac artery. We measured the longest length of the ureter from the inferior pole of the graft kidney to the tip of the ureter while trailing the ureter down to the kidney after completing the back-table procedures. The graft kidney was placed on the right iliac fossa and the iliac vessels were used for the anastomoses of the artery and vein. The vena cava or aorta was not used for the anastomosis in this cohort. During the study period, the donor and recipient surgeries were performed by the same surgical team, and no technical changes were made. In this study, two primary surgeons were involved in the recipient surgeries, which were performed or supervised by at least one of these surgeons. One primary surgeon performed or supervised all donor surgeries. Several surgeons, mostly residents, accompanied each surgery.

Ureterocystoneostomy was performed using the extravesical anastomosis method as previously reported (14). Briefly, the ureter was trimmed to the shortest length that was enough to reach the bladder, and the tip of the ureter was spatulated to 7 mm. The ureteroneocystostomy was conducted by the Lich–Gregoir method using 5-0 polydioxanone monofilament continuous sutures (15, 16). A 3 cm long submucosal tunnel was created as an anti-reflux procedure, and a 5-French 14 cm gauge double-J ureteral stent was placed. Urine was collected using a urethral catheter immediately after induction of anesthesia and before performing the surgery; sample for a urine culture was collected at the time of the catheter placement. Bladder capacity was measured by the free-fall water-filling method (upper limit 400 ml). We used cefazoline for donor nephrectomy and recipient surgery. The urethral catheter was removed on postoperative day 5, and the double-J catheter was removed using a cystoscope on postoperative day 6, unless any adverse events occurred. The voided volume after the removal of the double-J catheter was determined based on the bladder capacity measured in the operating room.

This study conformed with the principles outlined in the Declaration of Helsinki of 1964 and the Declaration of Istanbul of 2018. The protocol was approved by the ethics committee at Japanese Red Cross Kumamoto Hospital (study approval number 490), and the requirement of written informed consent was waived considering the retrospective and non-invasive nature of this study. None of the transplant donors were from a vulnerable population and all the donors or next of kin provided freely given written informed consent.

### Statistical Analysis

Baseline characteristics were evaluated for significant differences by Chi-square test for categorical variables, Shapiro–Wilk test of normality for quantitative variables, and t-test or Mann–Whitney U test for significant differences. Cox proportional hazard model was used to examine each factor that was considered to affect the incidence of UTI. A *p*-value <0.05 was considered to be significant. To identify independent predictors of outcomes, donor-related factors with significant differences were identified using univariate analyses, and multivariate analyses were performed with known factors, such as recipient sex, by using Cox proportional hazards models. Forward stepwise logistic regression was performed to identify the potential independent risk factors associated with the UTI within 5 years of transplantation. The analyses of the incidence of UTI within 5 years were performed using the Kaplan–Meier method, and statistical differences between curves were assessed using the log-rank test. Initial UTI events for recurrent cases were used for Kaplan–Meier and Cox analysis. Cases with missing data were not included in the study. All statistical analysis was performed using IBM SPSS Statistics for Windows, version 25 (IBM Corp., Armonk, NY, United States).

## Results

### Baseline Characteristics

Table 1 shows the baseline characteristics of the recipients and donors. The incidence of UTI within 5 years after transplantation was 13.7% (*n* = 29). Of these, 14 out of 29 recipients experienced recurrent UTIs (six UTIs: *n* = 2, five UTIs: *n* = 1, four UTIs: *n* = 1, three UTIs: n = 1, and two UTIs: *n* = 9). The median time of onset was 40.0 days after transplantation (IQR, 11.5∼445.5 days). There were six symptomatic uncomplicated UTI patients and 23 symptomatic complicated UTI patients. The distribution of the ureter length was not significantly different between the groups. Complications such as including uretero-ureteral anastomosis was not observed.

**TABLE 1:** Baseline characteristics.

Characteristics (*n* = 211)	UTI(−) *n* = 182 (86.3%)	UTI(+) *n* = 29 (13.7%)	*p* value
Recipient			
Recipient age, median (years old, IQR)	47.0 (33.0∼58.0)	52.0 (46.0∼61.0)	0.063
Female recipient, n (%)	51 (28.0%)	17 (58.6%)	0.001
Body weight, median (kg, IQR)	60.6 (52.0∼71.2)	59.0 (51.1∼67.4)	0.378
BMI, median (kg/m^2^, IQR)	22.2 (19.6∼25.0)	22.5 (19.9∼24.6)	0.863
BSA, median (m^2^, IQR)	1.7 (1.5∼1.8)	1.60 (1.5∼1.8)	0.232
Diabetes mellitus, n (%)	60 (33.0%)	12 (41.4%)	0.375
Dialysis dependence, n (%)	114 (62.6%)	21 (72.4%)	0.308
Duration of hemodialysis, median (months, IQR)	19.5 (7.8∼47.0)	26.0 (7.5∼77.0)	0.464
Bladder volume, median (mL, IQR)	304.6 ± 132.3	286.7 ± 146.3	0.508
Neurogenic bladder, n (%)	4 (2.2%)	1 (3.4%)	0.526
Recurrent UTI before transplantation, n (%)	3 (1.6%)	1 (3.4%)	0.449
Donor			
Donor age, median (years old, IQR)	57.0 (50.0∼64.3)	59.0 (53.5∼65.5)	0.332
Female donor, n (%)	127 (69.8%)	15 (51.7%)	0.054
Body weight, median (kg, IQR)	57.7 (51.6∼66.0)	61.0 (53.7∼67.3)	0.190
BMI, median (kg/m^2^, IQR)	22.7 (20.7∼24.9)	23.2 (22.0∼24.9)	0.388
BSA, median (m^2^, IQR)	1.6 (1.5∼1.7)	1.7 (1.5∼1.8)	0.138
Graft weight, median (g, IQR)	156.5 (136.0∼186.5)	168.0 (147.5∼226.0)	0.020
Graft major axis, median (mm, IQR)	105.0 (100.0∼110.0)	110.0 (100.0∼115.0)	0.126
Graft density (g/mm^3^, IQR)	1.2 (1.0∼1.6)	1.4 (1.1∼1.8)	0.286
Ureter length, median (cm, IQR)	11.5 (10.0∼12.0)	11.5 (11.0∼14.0)	0.080
Left kidney graft, n (%)	156 (85.7%)	29 (100.0%)	0.016
Recipient X Donor			
No. HLA mismatches (total), mean ± SD			
Class 1	2.0 ± 1.1	2.1 ± 1.0	0.538
Class 2	1.0 ± 0.6	1.1 ± 0.8	0.376
Total	3.0 ± 1.5	3.2 ± 1.6	0.378
Incompatible transplantation, n (%)	53 (29.1%)	13 (44.8%)	0.090

UTI, urinary tract infection; BMI, body mass index; BSA, body surface area; HLA, human leukocyte antigen.

For recipient-related factors, female sex (UTI vs. non-UTI: 58.6% vs. 28.0%, *p* = 0.001) was significantly different between the groups. There were no significant differences in age, weight, BMI, BSA, presence of diabetes mellitus, dialysis modality, duration of hemodialysis, and bladder capacity measured at the operating room. For donor-related factors, graft weight (UTI vs. non-UTI: 168.0 g [IQR: 147.5 g∼226.0 g] vs. 156.5 g [IQR: 136.0 g∼186.5 g], *p* = 0.020), and graft side (left, UTI vs. non-UTI: 100% vs. 85.7%, *p* = 0.016) were significantly different between the groups, while no significant differences were seen in age, sex, BW, BMI, BSA, major axis, and graft density. Other factors had no difference including the number of HLA mismatches and incompatible transplants. No patient had a history of catheterization before transplantation.

### Recipient Outcomes

Table 2 shows the recipient outcomes after transplantation, such as the duration of double-J stent, BK virus infection, post-operative complications. Of the 211 recipients, 22 experiences postoperative complications (one case of urinary leak, two cases of ureteral stenosis, one case of ureteral hemorrhage, five cases of lymphocele, two cases of hyper-acute rejection, five cases of hemorrhage, two cases of hematoma, one case of deep vein thrombosis, one case of duodenal ulcer, one case of acute respiratory distress syndrome, and one case of premature ventricular contraction with suspected cytomegalovirus myocarditis), and reoperations were performed in 11 cases. One patient required re-transplantation. There were no differences regarding graft and patient survivals between the groups.

**TABLE 2:** Recipient outcomes.

Characteristics (*n* = 211)	UTI(−) *n* = 182 (86.3%)	UTI(+) *n* = 29 (13.7%)	*p* value
5-year patient survival	98.4%	100%	0.640
5-year graft survival	93.4%	100%	0.161
All cause graft failure, n (%)	18 (9.9%)	2 (6.9%)	0.460
Creatinine at 1 year, median (mg/dL, IQR)	1.27 (1.02∼1.47)	1.11 (0.86∼1.42)	0.094
Urinary protein at 1 year, median (mg/day, IQR)	137.0 (79.0∼261.5)	88.0 (56.5∼195.5)	0.028
eGFR at 1 year, median (ml/min/1.73 m^2^, IQR)	47.4 (39.5∼53.8)	45.3 (38.1∼55.1)	0.947
ΔeGFR/year, median (ml/min/1.73 m^2^, IQR)	−0.64 (−1.85∼0.85)	−0.62 (−2.26∼1.01)	0.988
BK virus infection, n (%)	7 (3.8%)	2 (6.9%)	0.357
Rejection, n (%)	17 (9.3%)	1 (3.4%)	0.257
Post-operative complications, n (%)	20 (11.0%)	2 (6.9%)	0.389
Re-intervention, n (%)	11 (6.0%)	1 (3.4%)	0.489
Double-J stent (≧7 days), n (%)	32 (17.9%)	7 (25.0%)	0.255
Double-J stent placement duration, median (days, IQR)	6.0 (6.0∼6.0)	6.0 (6.0∼9.75)	0.118
*De novo* DSA, n (%)	23 (12.6%)	1 (3.4%)	0.123

UTI, urinary tract infection; GFR, glomerular filtration rate; DSA, donor specific antibody.

There were 39 recipients with long-term (more than 6 days) catheter placement, and there were no significant differences between the groups.

### Cox Proportional Hazard Model

Table 3 shows the results of the univariate and multivariate analyses of each factor by Cox proportional hazard model. In the univariate analysis, recipient sex (HR 3.16, 95% CI 1.51∼6.61, *p* = 0.002), graft weight (HR 1.12 per 10g, 95% CI 1.03∼1.20, *p* = 0.004), and ureter length (HR 1.27 per 1 cm, 95% CI 1.04∼1.55, *p* = 0.020) were significantly associated with UTI. Multivariate analysis that included donor sex revealed ureter length was an independent risk factor for UTI (HR 1.25 per 10mm, 95% CI 1.02∼1.53, *p* = 0.028), even after adjusting for the recipient sex (HR 3.05, 95% CI 1.45∼6.40, *p* = 0.003).

**TABLE 3:** Cox proportional hazard model.

Variable	Univariate analysis		Multivariate analysis	
	HR (95% CI)	*p value*	HR (95% CI)	*p value*
Recipient sex (ref. male)	3.16 (1.51∼6.61)	0.002	3.05 (1.45∼6.40)	0.003
Donor sex (ref. male)	0.53 (0.26∼1.09)	0.085		
Graft weight (per 10 g)	1.12 (1.03∼1.20)	0.004		
Ureter length (per 1 cm)	1.27 (1.04∼1.55)	0.020	1.25 (1.02∼1.53)	0.028

HR, hazard ratio; CI, confidence interval.

### Ureter Length and the Incidence of UTI

**FIGURE 1:**
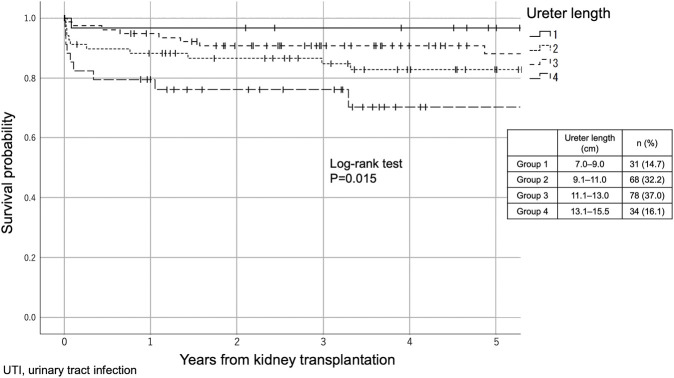
The difference in UTI incidence between the four groups according to ureter length (Kaplan–Meier method). The 211 recipients were stratified into the following four groups according to the ureter length: Group 1: 7.0–9.0 cm (*n* = 31, 14.7%), Group 2: 9.1–11.0 cm (*n* = 68, 32.2%), Group 3: 11.1–13.0 cm (*n* = 78, 37.0%) and Group 4: 13.1–15.5 cm (*n* = 34, 16.1%).

We stratified 211 recipients into the following four groups according to the ureter length; Group 1: 7.0∼9.0 cm (*n* = 31, 14.7%), Group 2: 9.1∼11.0 cm (*n* = 68, 32.2%), Group 3: 11.1∼13.0 cm (*n* = 78, 37.0%) and Group 4:13.1∼15.5 cm (*n* = 34, 16.1%). UTI-free survivals in 5 years were significantly different between the groups (*p* = 0.015, Log-rank test), and significant differences were observed between Groups 1 and 3 (*p* = 0.008) and between Groups 3 and 4 (*p* = 0.010, Figure 1).

## Discussion

UTI is one of the most frequent infections after kidney transplantation and is associated with acute rejection, allograft dysfunction, graft loss, and increased mortality (1, 3–6). Previous studies reported that older age, female recipient sex, history of UTI 1 month before kidney transplantation, recurrent UTI, congenital urinary tract malformations, VUR, ADPKD, and the number of days with an indwelling urinary catheter after kidney transplantation increase the incidence of UTI (8, 9). Differences in the anatomy of the urinary tract (short urethra and proximity of the urethral opening to the vagina and anus) are considered the reason for high risk of UTI in for women compared with that in men (6). Regarding age, elderly recipients, especially those over 65 years old, are at a higher risk of UTI due to decreased mobility, poor hygiene in nursing homes, a higher incidence of urinary retention by prostatic hyperplasia and bladder atrophy, and a weakened immune system (2, 3, 8, 9, 10, 11). There are contradictory reports regarding the role of DM; some studies have reported that it is involved in the incidence of UTI and while others have reported that it is not involved (3, 8, 17). Meanwhile, reported donor-related factors are limited to kidneys from deceased donors (6, 18). Chuang et al. assumed that a kidney from a deceased donor due to graft injury caused by prolonged ischemia time, or intense immunosuppressive drugs used for the induction of deceased donor kidney transplantation (6). As the availability of detailed deceased donor data was limited for privacy reasons in Japan, we could not include those cases for the analysis in the present study.

**FIGURE 2:**
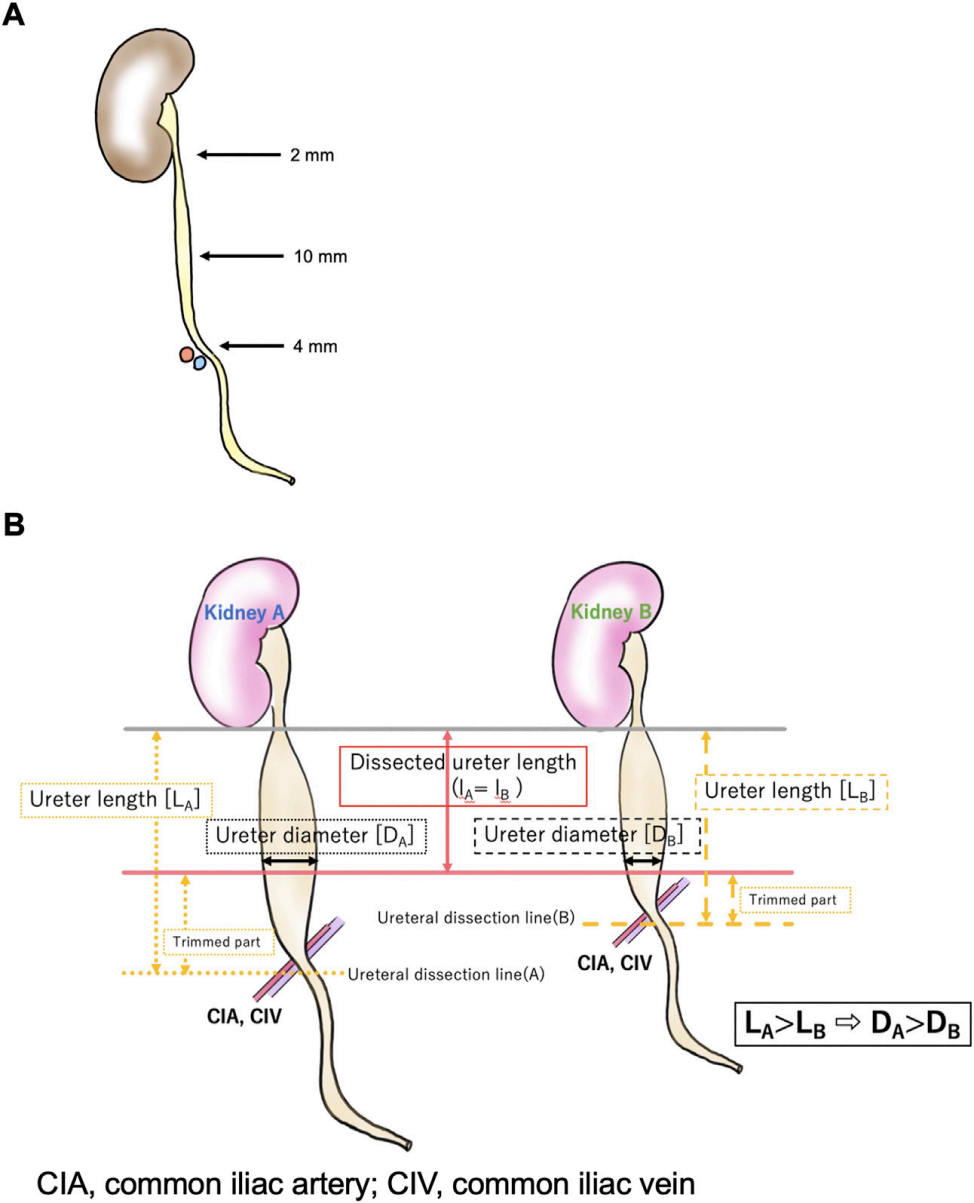
The relationships between the dissected ureter length and ureter diameter.

To our knowledge, this is the first study to propose the graft ureter length as a risk factor affecting UTI incidence in the long-term after living-donor kidney transplantation, even after adjusting for the well-known known factor, recipient female sex. Although the relation between graft ureter length and the incidence of UTI needs comprehensive discussion, we speculate that the length of the ureter is closely related to the diameter of the tip of the ureter trimmed for anastomosis. According to previous studies, the ureter tapers caudally from the pyeloureteral transition (5.67 ± 0.94 mm) to a small diameter (3.96 ± 0.65 mm) at the lower pole of the kidney. It then widens to its maximum diameter (5.11 ± 1.34 mm) at the abdominal ureter and retracts (3.59 ± 1.20 mm) at the pelvic margin across the common iliac artery(19, 20) (Figure 2A). The ureter was dissected at the lower level of the common iliac artery (median length of the removed ureter: 115.0 cm [95% CI, 100.0∼125.0 cm]) by the same surgeon, similar to the present study. Then the ureter was trimmed to the shortest length, enough to reach the bladder, and ureterocystoneostomy was performed using the extravesical anastomosis method by the same surgeons in a similar manner. Short ureters at the time of nephrectomy (before trimming) were anastomosed at the narrower diameter; the short ureter would hardly cause UTI in the standard technique. The distance from the iliac fossa, where the kidney was placed, to the bladder was approximately the same, regardless of body size; additionally, the length of the ureter after implantation was approximately the same. Thus, the long ureter is usually trimmed long (L) and ends up being used at the widest part (r) for anastomosis, which may increase the possibility of reflux from the bladder to the ureter in the standard technique (Figure 2B), as the large ureteral diameter is known to be one of the risk factors for severe UTI in VUR (21, 22).

Conversely, the duration of dialysis and preoperative bladder capacity, which are well-established risk factors for post-transplant VUR (23–25) were not associated with the frequency of UTI in the present study. According to a Japanese single-center report by Inoue et al., graft VUR was observed in 29.7% of recipients 1 year after living-donor kidney transplantation (24). Although we did not evaluate the VUR incidence after transplantation in the present study, the incidence of VUR was assumed to be similar as we adopted the same extravesical ureterocystoneostomy procedure. Given that asymptomatic bacteriuria occurs in 19–31% of recipients after kidney transplantation (26) and VUR is associated with increased post-voiding residual urine volume (27, 28), this high incidence of VUR can evoke febrile upper UTI. Thus, we included afebrile UTI in the lower urinary tract in the analysis.

Besides these factors, the long duration of indwelling urethral catheter insertion, age, and DM have been reported as risk factors (8, 9); however, no correlation was observed in our study. Regarding the duration of catheter insertion, removing the urethral catheter on postoperative day 5 and the double-J ureteral stent on postoperative day 6 in almost all cases might have contributed to no significant differences. The difference in UTI incidences between the present study and the other studies might be due to the relatively homogenous population. The frequency of HLA alleles varies by race and ethnicity, and island countries, such as Japan, exhibit a special genetic phenomenon called linkage disequilibrium in which a limited number of alleles are conserved as haplotypes (29). This allows a rather lower intensity of baseline immunosuppression than the US or Europe, where thymoglobulin is mainly used for the induction therapy (30–32).

There are several limitations to the present study. This is a single-center, retrospective study. We did not measure the length of the sacrificed ureter and the diameter of the ureter at the site of anastomosis. Also, we did not routinely check the post-transplant VUR. VUR may be a confounding factor for ureter length and ureter length may be a predictor of VUR. This aspect needs further investigation.

## Conclusion

The ureter length may be associated with the incidence of UTI after living-donor kidney transplantation. Further studies are needed to confirm the impact of ureter length and diameter on UTI incidence after living-donor kidney transplantation.

## Data Availability

The raw data supporting the conclusion of this article will be made available by the authors, without undue reservation.

## Abbreviations

ADPKD, autosomal dominant polycystic kidney disease; AMR, antibody-mediated rejection; AUC, area under the curve; BMI, body mass index; BSA, body surface area; BW, body weight; CFU, colony-forming unit; CI, confidence interval; CIA, common iliac artery; CIV, common iliac vein; CSA, cyclosporine; DSA, donor specific antibody; HLA, human leukocyte antigen; HR, hazard ratio; LDKT, living-donor kidney transplantation; MMF, mycophenolate mofetil; OR, operation room; TAC, tacrolimus; UTI, urinary tract infection; VUR, vesicoureteral reflex.
